# 1402. NTM Infections; A Rising Global Health Problem/Clinical Characteristics and Outcomes of Patients with Non-Tuberculous Mycobacterial Infections at Two Tertiary Academic Medical Centers

**DOI:** 10.1093/ofid/ofab466.1594

**Published:** 2021-12-04

**Authors:** Abdelhameed Nawwar, Julieta Madrid-Morales, Carolina Velez-Mejia, Rigoberto De Jesus Pizarro, Victor Cepeda, Kelly R Reveles, Jose Cadena-Zuluaga, Heta Javeri

**Affiliations:** 1 University of Texas Health Science Center at San Antonio, Texas, USA, San Antonio, Texas; 2 Southern Illinois Healthcare (SIH), Herrin, Illinois; 3 University of Texas at Austin, San Antonio, TX; 4 University of Texas health and science center San Antonio, Audie L. Murphy VA Medical Center, San Antonio, Texas; 5 UT Health San Antonio, San Antonio, Texas

## Abstract

**Background:**

Non-Tuberculous Mycobacteria (NTM) cause infections in immunocompetent as well as immunocompromised individuals affecting pulmonary and extra pulmonary sites. These pathogens are widely distributed globally and recent reports have shown their rise in many developed countries. Our study aimed to assess the disease magnitude, describe patient characteristics and risk factors, assess diagnostic and therapeutic measures and review outcomes furthering our understanding of the overall disease process.

**Methods:**

We conducted a retrospective, multicenter review of patients with positive NTM cultures treated at University Hospital System and South Texas Veterans Health Care System (STVHCS) from 2011 to 2018. Infections were classified as pulmonary or extrapulmonary, and we recorded demographics, microbiological data, treatment regimens, duration, complications, follow-up and mortality. All categorical variables were described using percentages and compared between groups using the chi-square test.

**Results:**

A total of 176 patients were included for analysis, of which 111 (63.1%) met criteria for NTM disease (2020 ATS/IDSA). The most common cultured mycobacterium was M. Avium Complex (MAC). M. abscessus-chelonae was more commonly associated with clinical disease and isolated from an extra pulmonary site whereas M. simiae complex had similar distribution between the infected and un-infected groups. Over 50% of patients received treatment (80% in the infected group). Cure was seen in 47.2%, all-cause mortality was 27% at last follow-up. Median duration of therapy was 10 months. 47% of patients experienced adverse effects which led to treatment discontinuation in one third of patients. Patients who were able to achieve a cure received a longer duration of therapy (12 vs 7 months; not statistically significant) and treatment was halted more commonly in the group that did not achieve eventual cure (42.6% vs. 16.7%, p=0.007).

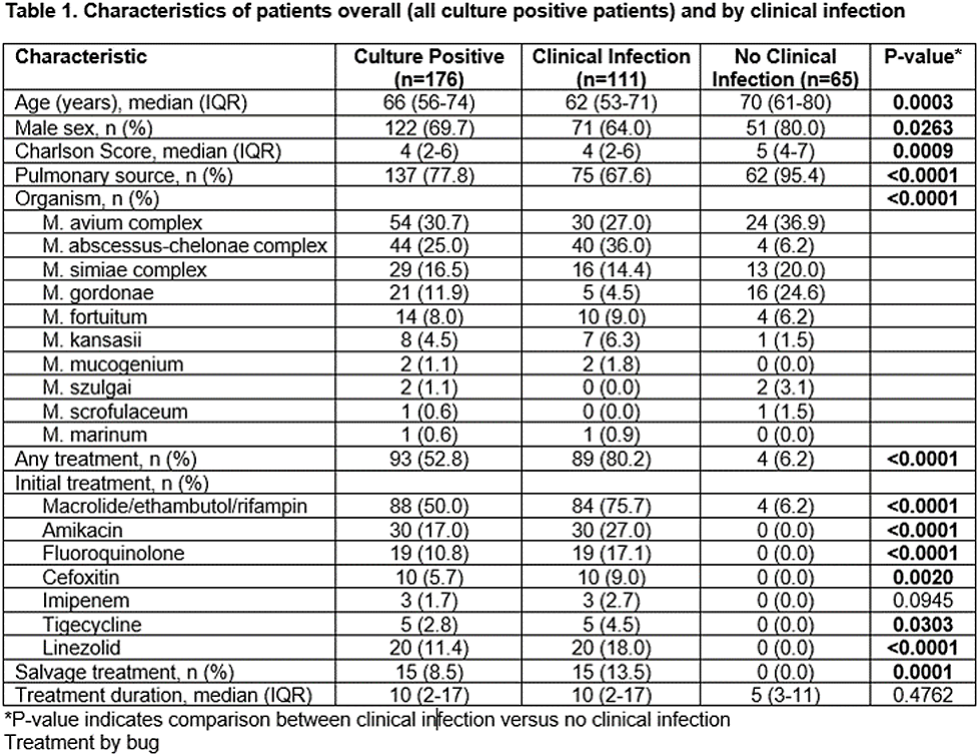

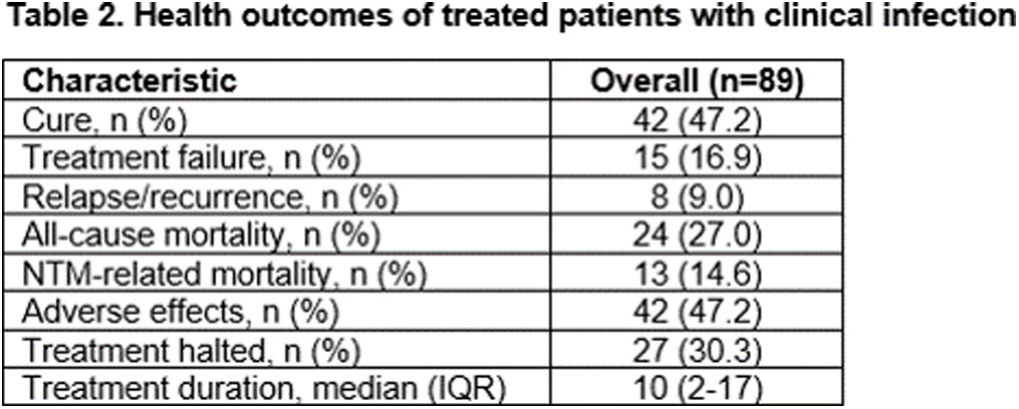

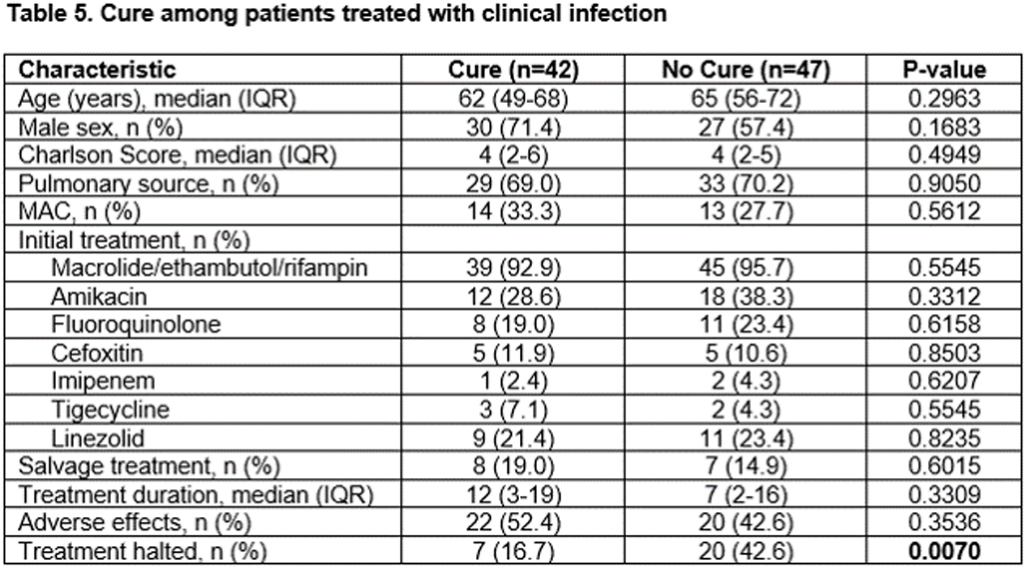

**Conclusion:**

NTM infections represent a therapeutic challenge with low cure rates and high mortality. An understanding of the risk factors, treatment options and outcomes is essential to guide appropriate management. Our study highlights high rates of adverse effects and discontinuation which precludes prolonged courses of therapy required to achieve cure.

**Disclosures:**

**All Authors**: No reported disclosures

